# Poor Oral Health as a Determinant of Malnutrition and Sarcopenia

**DOI:** 10.3390/nu11122898

**Published:** 2019-11-29

**Authors:** Domenico Azzolino, Pier Carmine Passarelli, Paolo De Angelis, Giovan Battista Piccirillo, Antonio D’Addona, Matteo Cesari

**Affiliations:** 1 Geriatric Unit, Fondazione IRCCS Ca’ Granda Ospedale Maggiore Policlinico, 20122 Milan, Italy; matteo.cesari@unimi.it; 2 Department of Clinical Sciences and Community Health, University of Milan, 20122 Milan, Italy; 3 Department of Head and Neck, Oral Surgery and Implantology Unit, Institute of Clinical Dentistry, Catholic University of Sacred Hearth, Fondazione Policlinico Universitario Gemelli, 00168 Rome, Italy; piercarminepassarelli@hotmail.it (P.C.P.); dr.paolodeangelis@gmail.com (P.D.A.); giovanbpiccirillo@gmail.com (G.B.P.); antonio.daddona@unicatt.it (A.D.)

**Keywords:** sarcopenia, nutrition, oral health, older people, malnutrition, swallowing, life course approach

## Abstract

Aging is accompanied by profound changes in many physiological functions, leading to a decreased ability to cope with stressors. Many changes are subtle, but can negatively affect nutrient intake, leading to overt malnutrition. Poor oral health may affect food selection and nutrient intake, leading to malnutrition and, consequently, to frailty and sarcopenia. On the other hand, it has been highlighted that sarcopenia is a whole-body process also affecting muscles dedicated to chewing and swallowing. Hence, muscle decline of these muscle groups may also have a negative impact on nutrient intake, increasing the risk for malnutrition. The interplay between oral diseases and malnutrition with frailty and sarcopenia may be explained through biological and environmental factors that are linked to the common burden of inflammation and oxidative stress. The presence of oral problems, alone or in combination with sarcopenia, may thus represent the biological substratum of the disabling cascade experienced by many frail individuals. A multimodal and multidisciplinary approach, including personalized dietary counselling and oral health care, may thus be helpful to better manage the complexity of older people. Furthermore, preventive strategies applied throughout the lifetime could help to preserve both oral and muscle function later in life. Here, we provide an overview on the relevance of poor oral health as a determinant of malnutrition and sarcopenia.

## 1. Introduction

Advancing age is characterized by a progressive decline in multiple physiological functions, leading to an increased vulnerability to stressors and augmented risk of adverse outcomes [1,2,3]. During the aging process, several factors may affect body shape from both clinical and functional perspectives. Reduction in smell and taste senses, poor appetite (the so-called “anorexia of aging”), and decreased energy expenditure may all contribute to poor nutrition. Moreover, illnesses, medications, as well as poor oral health (for example, due to teeth loss and poorly fitting dentures) can exacerbate anorexia [4,5,6]. Nutritional status among older people may be also influenced by living or eating alone, poor financial status, dismobility, and decreased ability to shop or prepare meals [7,8]. Psychosocial factors including loneliness, sleep disorders, dementia, and depression are also recognized to have a negative impact on the dietary intake of older subjects [9]. 

Furthermore, with aging, there is a progressive loss in muscle mass and strength, whereas fat mass and fat infiltration of muscle increase [10,11]. Sarcopenia is the term, introduced for the first time in 1988 by Irwin Rosenberg, to indicate the pathologic reduction in muscle mass and strength leading to a poor function [12,13]. Interestingly, in recent years, it has been highlighted that sarcopenia is not limited to lower limbs, but is a whole-body process [14,15,16], also affecting the muscles devoted to chewing and swallowing [10,17], with a negative impact on food intake. In fact, atrophy of muscles critical for the respiratory and swallowing functions has been reported [14,18,19,20,21,22]. 

The variety of dental problems experienced by older people can result in chewing difficulties determining changes in food selection, thus leading to malnutrition and consequently to frailty [23] and sarcopenia [10,23]. Poor oral status may also predispose one to a chronic low-grade systemic inflammation through periodontal disease [24,25], which has an increased prevalence in those who are not able to perform the daily oral hygiene procedures [26], and it is a well-known risk factor in the pathogenesis of frailty [27] and sarcopenia [28]. Furthermore, periodontal disease has been associated with faster decline in handgrip strength [29], and recent studies showed an association between chewing difficulties and frailty [24]. 

**Figure 1:**
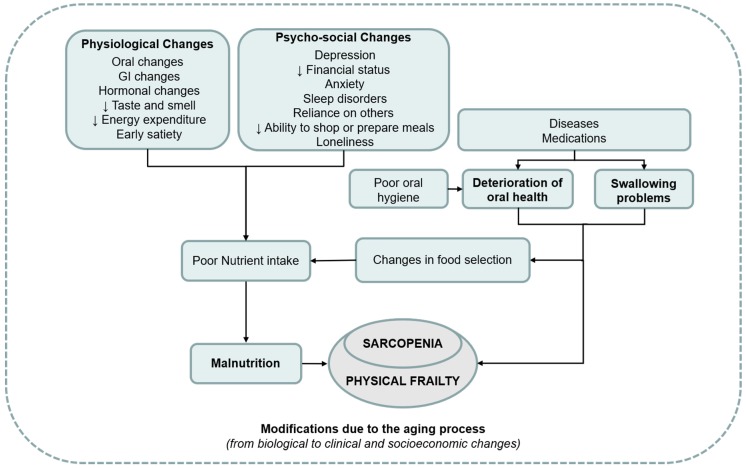
Overview of the interplay between poor oral status, malnutrition, and sarcopenia. GI—gastrointestinal.

Therefore, a hypothetical triangle oral status–nutrition–sarcopenia, exposing the older person to the frailty disabling cascade, may be suggested, as seen in Figure 1. 

## 2. Oral Changes with Aging

Poor oral health is not an inevitable part of aging since good care throughout the life course can result in the maintenance of functional teeth later in life [24]. Throughout a lifetime, the oral cavity experiences a variety of physiological modifications, such as enamel changes, fractures lines and stains, as well as dentin exposure and darkening of the tooth. At the same time, in the inner part of the tooth, several changes, such as the deposition of secondary dentin reducing the size of the pulp chamber and canals, may also occur [30]. Furthermore, in older people, tooth wear is frequently observed, affecting more than 85% of all the teeth groups in both the mandible and maxilla [31]. Additionally, a loss in terms of elastic fibers in the connective tissue has been documented and, subsequently, the oral mucosa becomes less resilient [32].

However, older people, especially those who are institutionalized or with limited financial resources, may experience problems to access oral care. Furthermore, it has been documented that older people frequently have difficulty expressing complaints and assign low priority to oral health until dental problems become intolerable [33]. Oral problems among older people have been implicated in a high prevalence of tooth loss, dental caries, periodontal disease, xerostomia, and oral precancer/cancer lesions [34]. Periodontitis and dental caries are very common diseases, especially in older people, and are considered the main cause of tooth loss [35].

Around the age of 70, there is also a peak of root/cementum caries, as a result of both tooth retention and major exposure of these surfaces following periodontal support loss. Moreover, older people are at higher risk of periodontitis since it is a cumulative disease, especially with regard to the multirooted teeth [36]. 

### 2.1. Edentulism

Edentulism is a pathological condition characterized by multiple missing teeth; it can be partial or total. The etiology of tooth loss includes factors such as predisposition, diet, hormonal status, coexisting diseases, hygiene habits, and use of dental clinics. Additionally, edentulism may result from an unsuccessful periodontal treatment or important carious lesions [37,38]. Dental disease and loss of teeth are not part of normal aging, but if this occurs, it is probably a result of neglected oral hygiene and/or an inadequate treatment [39,40]. Edentulism is exacerbated when masticatory function is not restored with dental prostheses [41]. Tooth loss affects the individual ability to chew determining an alteration of food choices [42]. Indeed, edentulous people are at greater risk of malnutrition than dentate or partially dentate individuals [43], and, consequently, with an increased susceptibility to sarcopenia and frailty [25]. Tooth loss is also a risk factor for disability, since it impedes self-sufficiency and worsens the quality of life [42]. 

### 2.2. Dry Mouth

Saliva is pivotal for bolus formation and consequently is also related to the sensory and textural experience. Xerostomia is a clinical condition characterized by an excessive sensation of dryness in the mouth, which is not necessarily linked to salivary gland hypofunction [30,44]. Xerostomia is estimated to affect 25–50% of older individuals [45]. Etiologic factors include polypharmacy (especially with antihypertensives, antidepressants, and antipsychotics) [46], diseases, poor general health, female sex, and older age [47,48]. Furthermore, radiation for head and neck cancers can damage salivary glands, leading to permanent xerostomia [49]. With aging, there is also a reduced salivary flow in salivary glands, which cannot be explained only on the basis of medications [50]. In fact, salivary hypofunction and xerostomia are two distinct constructs that are frequently improperly used interchangeably [33].

However, it has been reported that nearly one third of older adults complaining of xerostomia do not present any reduction of the salivary flow or saliva secretion. This suggests a psychological component may be involved when reporting the symptom [30]. Nonetheless, hyposalivation may seriously compromise chewing function and early digestive process. A reduced quantity of saliva can, in fact, affect the preparation of the alimentary bolus and the swallowing [51]. 

### 2.3. Periodontal Disease

Periodontitis is described as a chronic inflammatory disease that affects the supporting tissues of the teeth, leading to a progressive destruction of the periodontium [52]. It can also cause mobility and displacement of the remaining teeth and is often linked to difficulty in chewing. Prevalence of periodontal disease, considering a periodontal index score of 4 (deep pockets), ranges from approximately 5% to 70% among older people [53]. Periodontitis is a cumulative disease; therefore, it becomes increasingly severe as the person ages [30]. Poor oral hygiene is a critical determinant of periodontitis since it leads to the formation of dental plaque containing microorganisms [54]. Systemic risk factors for periodontal disease also include other behaviors, such as smoking, medical conditions (i.e., poorly controlled diabetes, obesity, stress, osteopenia), and inadequate dietary consumption of calcium and vitamin D [55]. Since periodontitis share some characteristics with other systemic inflammatory diseases, a relationship between periodontitis and other inflammatory pathologies (i.e., diabetes, cardiovascular diseases, adverse pregnancy outcomes, and rheumatoid arthritis) has been proposed [56]. 

In recent years, the role of the diet in periodontitis has been highlighted. To date, it has been documented that a diet poor in fruit and vegetables and therefore in micronutrients may lead to a greater inflammatory response of periodontal tissues that support the tooth. Interestingly, a recent systematic review of the relationship between dietary intake and periodontal health in community-dwelling older adults, reported positive associations between periodontal disease and lower intakes of docosahexaenoic acid, vitamin C, vitamin E, β-carotene, milk, fermented dairy products, dietary fiber, fruits and vegetables, and higher intakes of omega-6/omega-3 ratio and saturated fatty acids [57]. Additionally, micronutrient deficiencies can negatively affect healing following periodontal surgery [58]. At the same time, the loss of dental elements due to periodontitis can negatively affect the nutritional status of the patient, resulting in a discomfort during chewing and leading to a selection of soft and easy-to-chew foods.

### 2.4. Dental Caries

Dental caries is a multifactorial infectious disease characterized by the demineralization and destruction of the dental substance: enamel, in fact, is susceptible to acid dissolution over time. The pathological changes of the dental structure may have serious consequences, ultimately leading to the breakdown of the teeth themselves [59]. The prevalence of dental caries varies between 20% and 60% in community-dwelling older people and 60% and 80% in care home settings [60,61,62,63,64]. Various predisposing conditions to dental caries have been reported, including carbohydrate (especially simple sugars) consumption, diabetes, and poor socioeconomic conditions [60,65,66,67,68].

With increasing age, people may experience physical and cognitive decline, which may result in poor oral hygiene, leading to an increased incidence of caries. Over time, small lesions already filled can need a larger dental restoration, that can lead to a tooth fracture or an endodontic treatment [30]. Endodontic therapy (also known as root canal treatment) is a necessary procedure in case of inflamed or infected dental pulp. It consists in the removal of the pulp, both in the coronal and radicular part of the tooth, and in its replacement with a gutta-percha permanent filling (a substance of vegetable origin such as natural rubber). Xerostomia is closely related to a higher risk for developing caries since loss of saliva may lead to an increased acidity of the mouth. This leads to different situations that may contribute to the development of the dental caries: the proliferation of bacteria, the loss of minerals from the tooth surfaces, and the loss of lubrication [69]. 

### 2.5. Impact of Oral Health on Nutritional Status

Nutrition is a key modulator of health in older persons. Inadequate intake of nutrients is a well-known contributing factor in the progression of many diseases. This also has a significant impact in the complex etiology of sarcopenia and frailty [70,71,72]. Due to a decline in many functions, including poor oral status, dietary intake is often compromised in older people and the risk of malnutrition is increased. Particularly, acute and chronic illnesses and medications as well as poor dentition can exacerbate anorexia [5,70,73]. Oral problems in older individuals are associated with modifications in food selection and, therefore, in nutrient intake [25]. Deterioration of oral health can ultimately lead to the development of chronic conditions such as diabetes [74] and cardiovascular problems [75,76,77]. Masticatory performance is affected by the number of teeth in functional occlusion [78,79,80], the maximal biting force [81,82], denture wearing [83] and xerostomia [84]. The functional occlusion during mandibular closure is provided by the even and simultaneous contact of all remaining teeth (at least 20 with 10 contiguous teeth in each arch) [85].

Tooth loss has been implicated in the reduction of chewing ability and in difficulties in bolus formation [86]. To date, it has been reported that as number of remaining teeth decrease, the bolus size increases leading to a dysfunctional swallowing [87]. Edentulous individuals, even when using well-made dentures, may experience more chewing difficulties than dentate people [88]. Therefore, they may be considered as the group more prone to changing their diet [89,90]. Older people who experience dental problems frequently avoid harder foods such as meats, fruits, and vegetables which are typically major sources of proteins, fiber, vitamins, and minerals [41,88,91]. The lack of these latter key nutrients may expose older individuals to an increased risk for malnutrition, frailty, and sarcopenia [24,92]. In addition, it is well established that micronutrient deficiencies, even subtle, may lead to oxidative stress and consequently to inflammation. Therefore, these processes can further exacerbate sarcopenia and frailty and become a clear risk factor for periodontitis. Nutritional deficiencies may also negatively affect the mineralization process, increasing the susceptibility to dental caries [93]. Furthermore, undernutrition can exacerbate the severity of oral infections [94]. Indeed, with advancing age, people show a tendency to select soft foods due to difficulty and fatigue of chewing [10,95]. However, these latter are frequently processed foods that are high in fat and sugar and with a poor content of vitamins and minerals, leading to fat deposition, oxidative stress, inflammation, and, consequently, increased risk of cardiovascular disease and metabolic syndrome [88,95,96,97]. In fact, it is well established that obesity leads to chronic low-grade inflammation, increasing the susceptibility to dental caries, periodontal disease, and tooth loss [98]. The excess of energy is stored in adipocytes and leads to both hypertrophy and hyperplasia, resulting in an abnormal adipocyte function. This may increase mitochondrial stress and altered endoplasmatic reticulum function. Furthermore, adipocyte-associated inflammatory macrophages can also induce oxidative stress [99]. On the other hand, it is widely recognized that an excessive consumption of simple sugars is a major risk factor for dental caries [100,101]. 

Large epidemiological studies, such as the UK National Diet and Nutrition Survey (NDNS) [102] and the US National Health and Nutritional Examination Surveys (NHANES) [103,104], reported an association between poor dental status and inadequate dietary intake in older people. In particular, they reported that edentulous subjects, with and without prosthesis, consumed less fruits and vegetables. Moreover, decreased protein and micronutrient intake, together with increased carbohydrate consumption, has been reported in people with less than 21 teeth [104]. 

## 3. Sarcopenia and Oral Status 

Sarcopenia, defined as the progressive and accelerated loss of muscle mass and function, is a major determinant of several adverse outcomes including frailty, disability, and mortality [13,105]. Although sarcopenia is a condition commonly observed with the aging process, it can also occur earlier in life [106]. Since 2016, sarcopenia has been recognized as an independent condition with an International Classification of Disease, 10th Revision, Clinical Modification (ICD-10-CM) Diagnosis Code [107]. Recently, the European Working Group on Sarcopenia in Older People (EWGSOP) [106] updated their consensus on definition and diagnosis (EWGSOP2). In this revised consensus, low muscle strength is considered a key characteristic of sarcopenia, and poor physical performance is identified as indicative of severe sarcopenia. Moreover, EWGSOP2 have recommended specific cut-off points to identify and characterize the sarcopenic condition, and provide an algorithm that can be used for case-finding.

Sarcopenia has a complex multifactorial pathogenesis, which involves lifestyle habits (i.e., malnutrition, physical inactivity), disease triggers, and age-dependent biological changes (i.e., chronic inflammation, mitochondrial abnormalities, loss of neuromuscular junctions, reduced satellite cell numbers, hormonal alterations) [108,109]. Sarcopenia is a whole-body process, affecting not only lower extremities, but also muscles dedicated to breathing, mastication, and swallowing [14,18,19,20,21,22]. In particular, swallowing is a complex mechanism involving several head and neck muscles simultaneously and in conjunction to coordinate the entire process [110]. Several age-related changes, such as as reduction of tissue elasticity, changes of the head and neck anatomy, reduced oral and pharyngeal sensitivity, and impaired dental status, may contribute to different degrees to a subtle swallowing impairment, the so called “presbyphagia”. It is usually an asymptomatic condition in which swallowing function is preserved, but tends to slowly worsen as the aging process advances [16,111]. Presbyphagia may increase the risk of dysphagia and aspiration in older people, especially during acute illnesses and other stressors [112]. Moreover, reductions in muscle mass of the geniohyoid, pterygoid, masseter, tongue, and pharyngeal muscles have been documented in older individuals [20,113,114,115]. Several authors also reported a decline in the strength of the swallowing muscles with aging or sarcopenia [116]. Maximal tongue strength decreases with aging [116,117,118,119], and there is some evidence that aging leads to a decreased jaw-opening force in older men. Several authors also reported an association between tongue strength and handgrip strength [120,121]. A decrease in tongue strength has been associated with a decline of activities of daily living [122], and a reduced tongue thickness has been noted in people with low body weight [20]. 

Lip function is also important for feeding. In fact, poor lip muscle closure may cause leakage through the corners of the mouth [123]. Additionally, decreased lip strength has been suggested to occur due to sarcopenia and to be related to difficulties in eating and drinking (i.e., dysphagia) [117]. Lip force has been associated with hand grip strength and lip pendency has been associated with aging [117,124].

Indeed, since it has been shown that skeletal muscle mass and strength decline may affect both swallowing and general muscle groups, a new condition, called “sarcopenic dysphagia” has been coined [22,124,125]. Swallowing muscles are characterized by a high percentage of type II fibers, which are more easily affected by malnutrition and sarcopenia than type I muscle fibers [22]. However, some cranial muscles, including the jaw-closers, are very different in fiber-type composition than other skeletal muscle groups (i.e., limbs or abdomen). For instance, the masseter muscle, which originates from the zygomatic arch, contains both type I and type II fibers, but shows a predominance of type I muscle fibers, which are more strongly affected by inactivity rather than aging [126,127]. Given that the meal texture of older people frequently becomes softer, less power of tongue movement and of masseter muscle is required, which may result in decreased activity of these muscles.

Interestingly, poor oral health may predispose one to a chronic low-grade inflammatory state through periodontal disease, which is a well-known risk factor for frailty and sarcopenia [25,128,129]. In fact, the detrimental effects of periodontitis are not confined solely to the oral cavity, but extend systemically, leading to metabolic alterations [130], including insulin resistance [131], diabetes [131,132], arthritis [133], and heart disease [134]. Furthermore, alterations in mitochondrial function leading to oxidative stress through the production of reactive oxygen species (ROS) have also been reported to mediate both oral and systemic pathologies (i.e., sarcopenia) [108,135,136,137]. Given their regulatory role as signaling molecules in autophagy, it has been speculated that elevated ROS production in periodontal disease could lead to autophagic alterations [138]. Bullon et al. [139] found high levels of mitochondrial-derived ROS, accompanied by mitochondrial dysfunction in peripheral blood mononuclear cells from patients with periodontitis. Moreover, oral gingiva seems to be highly responsive to the lipopolysaccharides (LPS), which are bacterial endotoxins prevalent in periodontal disease. In fact, gingival fibroblasts, which play an important role in remodeling periodontal soft tissues, may directly interact with LPS. In particular, LPS from *Porphyromonas gingivalis* enhances the production of inflammatory cytokines [140]. *Porphyromonas gingivalis* has been found to be responsible for high mitochondrial ROS and coenzyme Q10 levels, and for mitochondrial dysfunction, given its influence on the amount of respiratory chain complex I and III [138,139]. Indeed, LPS-mediated mitochondrial dysfunction could explain the oxidative stress onset in patients with periodontitis. Furthermore, Hamalainen et al. [29] reported an association between periodontitis and quicker declines in handgrip strength. 

On the other hand, as discussed in the previous section, the variety of dental problems experienced by older people can lead to a decline in general health through poor nutrient intake, pain, and low quality of life [25]. Poor oral status has been reported to affect 71% of patients in rehabilitation settings [141] and 91% of people in acute-care hospitals [142], and has been associated with malnutrition, dysphagia, and reduced activities of daily living [17]. Hence, poor oral status may lead to sarcopenia through poor nutrient intake. Moreover, inflammation further contributes to malnutrition through various mechanisms, such as anorexia, decreased nutrient intake, altered metabolism (i.e., elevation of resting energy expenditure), and increased muscle catabolism [143]. Chronic inflammation is a common underlying factor, not only in the etiology of sarcopenia, but also for frailty. In fact, sarcopenia and frailty are closely related and show a remarkable overlap especially in the physical function domain [144,145,146]. The presence of oral problems, alone or in combination with sarcopenia, may thus represent the biological substratum of the disabling cascade experienced by many frail individuals.

## 4. Interventions

The management of older people should be multimodal and multidisciplinary, especially for those with or at risk of malnutrition [147], in order to improve different conditions (i.e., oral problems and sarcopenia). From a practical point of view, comprehensive geriatric assessment (CGA) is the multidimensional, interdisciplinary diagnostic and therapeutic process aimed at determining the medical, psychological, and functional problems of older people. The CGA’s objective is the development of a coordinated and integrated plan for treatment and follow-up in order to maximize overall health with aging [148]. To date, increasing evidence suggests that prosthodontic treatment in combination with personalized dietary counselling may improve the nutritional status of patients [51]. Here, we provide an overview on the management of oral problems, malnutrition, and sarcopenia. 

### 4.1. Oral Management

The stomatognathic system is very vulnerable over time, but with special care, it can be preserved throughout the lifetime [30]. Nevertheless, one of the major challenges in providing both restorative and preventive care for older adults is to check dental status on a regular basis [34]. Prevention is pivotal to detecting oral disease as soon as possible and requires regular patient contact. However, since it has been reported that older people frequently fail to achieve a good oral hygiene, both patients and caregivers should be made more aware about the importance to check dental status as well as oral hygiene. 

The oral health-care professionals should develop a personalized program, in order to prevent all the problems related to the aging process. In some cases, it is difficult to provide dental care in the hospital setting in a short time, since in many countries there are long waiting lists (especially in publicly funded hospitals) [149]. Therefore, private dentists also need better awareness concerning the complexity of older people. There is, first and foremost, a need to understand the level of dependency, the medical condition, and the physical or cognitive impairment of the patient. Secondly, it is important to establish an oral healthcare plan that includes both professional and self-care elements [150].

The oral management of older people usually involves different aspects:

(1) For the teeth affected by carious lesions, it must be recommended that prompt treatment be provided in order to prevent tooth loss. It would be equally appropriate for endodontic treatments for teeth with endodontic problems.
(2) It is very important to monitor the periodontal status of the older patient and to provide a proper treatment plan, such as modification of general health-risk factors and oral health-specific risk factors, but professional hygiene or surgical procedures may also be necessary.
(3) Prosthetic rehabilitation of the edentulous patient may help to prevent malnutrition [151] since it restores the chewing function.
(4) In order to prevent problems related to the xerostomia and reduce exacerbation of carious lesions, it may be helpful to treat with saliva substitutes.

### 4.2. Nutritional Interventions

As discussed above, nutrition is an important determinant of health in older people. Thereby, it is pivotal to provide adequate amounts of energy, proteins, fluid, and micronutrients in order to prevent or treat excess or deficiencies, and therefore improve several health-related outcomes in terms of morbidity and mortality. A personalized approach is pivotal in order to respect individual preferences, needs, and to increase compliance to the diet. Nutritional status should be assessed before each intervention, and the amount of energy and proteins should be individually adjusted with regard to nutritional status, physical activity level, disease status, and tolerance [152]. The European Society for Clinical Nutrition and Metabolism (ESPEN) [152], in its guidelines on clinical nutrition and hydration in geriatrics, recommends a guiding value for energy intake of 30 kcal/kg of body weight/day. However, as stated above, it should be adapted individually. Both ESPEN [153] and the PROT-AGE study group [147] recommend providing a protein intake of at least 1.0 g/kg body weight/day in older people to maintain muscle mass, increasing the intake up to 1.2–1.5 g/kg body weight/day in presence of acute or chronic illness. Additionally, it seems that the per-meal anabolic threshold of protein intake is higher in older individuals (i.e., 25 to 30 g protein/meal, containing about 2.5 to 2.8 g leucine) than young adults [147]. However, since older people may experience difficulty of ingesting large amounts of proteins in a single meal, supplementation should be considered. Since serum vitamin D levels decline gradually with aging [154,155] and have been associated with reduced muscle mass and strength, supplementation should thus be considered in those who are deficient. 

Food texture should be adapted depending on the chewing and swallowing condition in order to avoid choking risk [10]. Harder foods may be modified to soft consistencies (i.e., bite-sized, minced, pureed) requiring little chewing, as well as liquids, which may be thickened to render the swallowing process slower and safer [10,156,157]. Controlling the intake of simple sugars is pivotal to prevent both dental caries [101] and metabolic complications [158]. World Health Organization recommends to limit the intake of free sugars to less than 10% of total energy intake to minimize the risk of dental caries [159]. 

Fruit and vegetables are major sources of minerals and vitamins with antioxidant properties; therefore, their consumption should be promoted both for oral and general health. It has been documented that excessive antioxidant supplementation could compromise both the mechanism of adaption to exercise and have even pro-oxidant effects. Thus, supplementation in people who are not deficient should be regarded carefully [160]. Dietary consumption of fatty fish (i.e., salmon, mackerel, herring, lake trout, sardines, albacore tuna, and their oils), which are a major source of omega-3 fatty acids, has been associated with a greater fat-free mass [161]. Given their antioxidant role, omega-3 fatty acid supplementation has been suggested to improve inflammatory status both in periodontal disease [162] and sarcopenia [163]. However, more studies are needed to further elucidate the exact time and dosage of supplementation as well as long term effects [164]. Nevertheless, consumption of foods rich in omega-3, such as as fatty fish, should be promoted. 

### 4.3. Exercise and Rehabilitative Strategies

Physical inactivity is considered one of the main causes of sarcopenia [165] because it determines a resistance to muscle anabolic stimuli [166]. Moreover, it has been proposed that physically inactive individuals may have a greater risk of periodontal disease [167]. In particular, resistance training seems to be the most effective type of exercise to counteract sarcopenia [168]. Furthermore, since sarcopenia is a systemic process [15,21], it has been recommended to perform a holistic training involving all muscle groups [15]. In fact, it has been documented that both masticatory and swallowing functions can be improved through muscle-strengthening exercises [169,170]. Several studies reported enhancements in subjective chewing ability, swallowing function, salivation, relief of oral dryness, and oral-health quality of life. Indeed, the synergistic effect of nutritional interventions coupled with physical exercise may improve both muscle [164] and oral health [167]. Recently, Kim et al. [171] reported an improvement in oral function following an exercise program which included stretching of the lip, tongue, cheek, masticatory muscle exercise, and swallowing movements. Several studies have been focused on swallowing rehabilitation. To date, a positive effect of expiratory muscle resistance training has been documented in improving suprahyoid muscle activity [172,173]. Furthermore, head lift exercises showed a beneficial impact on swallowing movements [174,175], and tongue strengthening exercises have been reported to enhance tongue strength [176,177]. Yeates et al. [178] demonstrated that isometric tongue strength exercises and tongue pressure accuracy tasks improved isometric tongue strength, tongue pressure generation accuracy, bolus control, and dietary intake by mouth. It has also been reported that tongue exercises prevented general sarcopenia [178,179]. Indeed, swallowing muscles training, despite its focus on swallowing function, may exert its beneficial effects systemically. 

## 5. Conclusions

Aging is characterized by a progressive loss of physiological integrity, leading to a decline in many functions and increased vulnerability to stressors. Many changes in masticatory and swallowing function are subtle but can amplify disease processes seen with aging. Nevertheless, it is often difficult to clearly distinguish the effects of diseases from the underlying age-related modifications. Several stressors, including oral problems, may therefore negatively impact on the increasingly weak homeostatic reserves of older individuals. As a healthy diet may have a systemic beneficial effect, oral care also shows an important role in maintaining and improving not only oral health, but also general health and well-being. 

Overall, severe tooth loss, as well as swallowing and masticatory problems, partly contribute to restricted dietary choices and poor nutritional status of older adults, leading to frailty and sarcopenia. On the other hand, oral diseases might be influenced both by frailty and sarcopenia, probably through biological and environmental factors that are linked to the common burden of inflammation and oxidative stress. 

A multidisciplinary intervention of dental professionals, geriatricians, nutritionists, and dietitians may help to provide better care and preserve the functional status of older people. Increasing evidence also suggests that oral care, when offered with personalized nutritional advice, may improve the nutritional status of patients. A life course approach to prevention at a younger age, including diet optimization and oral preventive care, as well as physical activity, may help in preserving both oral and muscle function later in life.
